# NSDHL contributes to breast cancer stem-like cell maintenance and tumor-initiating capacity through TGF-β/Smad signaling pathway in MCF-7 tumor spheroid

**DOI:** 10.1186/s12885-024-13143-3

**Published:** 2024-11-08

**Authors:** So-Hyun Yoon, Sangeun Lee, Hoe Suk Kim, Junhyuk Song, Moonjou Baek, Seungyeon Ryu, Han-Byoel Lee, Hyeong-Gon Moon, Dong-Young Noh, Sangyong Jon, Wonshik Han

**Affiliations:** 1 Department of Surgery, Seoul National University College of Medicine, Seoul, 03080 Republic of Korea; 2 Cancer Research Institute, Seoul National University, Seoul, 03080 Republic of Korea; 3 Biomedical Research Institute, Seoul National University Hospital, Seoul, 03080 Republic of Korea; 4 Interdisciplinary Programs in Cancer Biology Major, Seoul National University Graduate School, Seoul, 03080 Republic of Korea; 5 Integrated Major in Innovative Medical Science, Seoul National University Graduate School, Seoul, 03080 Republic of Korea; 6 Genomic Medicine Institute, Medical Research Center, Seoul National University, Seoul, 03080 Republic of Korea; 7 Center for Precision Bio-Nanomedicine, Department of Biological Sciences, Korea Advanced Institute of Science and Technology (KAIST), Daejeon, 34141 Republic of Korea

**Keywords:** NSDHL, MCF-7 human breast cancer cell, Tumor spheroid, Cancer stem cell, Cholesterol, TGF-β

## Abstract

**Background:**

NAD(P)-dependent steroid dehydrogenase-like protein (NSDHL), which is involved in breast tumor growth and metastasis, has been implicated in the maintenance of cancer stem cells. However, its role in regulating breast cancer stem-like cells (BCSCs) remains unclear. We have previously reported the clinical significance of NSDHL in patients with estrogen receptor-positive (ER +) breast cancer. This study aimed to elucidate the molecular mechanisms by which NSDHL regulates the capacity of BCSCs in the ER + human breast cancer cell line, MCF-7.

**Methods:**

NSDHL knockdown suppressed tumor spheroid formation in MCF-7 human breast cancer cells grown on ultralow-attachment plates. RNA sequencing revealed that NSDHL knockdown induced widespread transcriptional changes in the MCF-7 spheroids. TGF-β signaling pathway was the most significantly enriched Kyoto Encyclopedia of Genes and Genomes (KEGG) pathway (fold change ≥ 2, *P* ≤ 0.05) identified in NSDHL-knockdown MCF-7 spheroids compared with the control. In orthotopic tumor models injected with NSDHL-knockdown MCF-7 spheroids, tumor initiation and growth were strongly suppressed compared with those in the control.

**Results:**

BCSC populations with CD44+/CD24- and CD49f+/EpCAM + phenotypes and high ALDH activity were decreased in NSDHL-knockdown MCF-7 spheroids and xenograft tumors relative to controls, along with decreased secretion of TGF-β1 and 3, phosphorylation of Smad2/3, and expression of SOX2. In RNA-sequencing data from The (TCGA) database, a positive correlation between the expression of NSDHL and SOX2 was found in luminal-type breast cancer specimens (*n* = 998). Our findings revealed that NSDHL plays an important role in maintaining the BCSC population and tumor-initiating capacity of ER-positive MCF-7 spheroids, suggesting that NSDHL is an attractive therapeutic target for eliminating BCSCs, thus preventing breast cancer initiation and progression.

**Conclusions:**

Our findings suggest that NSDHL regulates the BCSC/tumor-initiating cell population in MCF-7 spheroids and xenograft tumors.

**Supplementary Information:**

The online version contains supplementary material available at 10.1186/s12885-024-13143-3.

## Background

In the treatment of estrogen receptor-positive (ER+) breast cancer, Reducing the risk of late relapse remains a significant oncological challenge in the treatment of ER-positive breast cancer. Recurrence is thought to be caused by breast cancer stem cells (BCSCs) with CD44+/CD24- surface markers and high ALDH activity [1–3]. In clinical and epidemiological studies, high cholesterol levels have been associated with the onset of resistance to tamoxifen therapy, an increased risk of recurrence, and accelerated tumor initiation and growth in patients with ER + breast cancer [4–8]. However, the molecular mechanisms underlying the risk of ER + breast cancer in patients with elevated cholesterol levels remain unclear.

Cholesterol accumulation is an accelerator of cancer development and progression in diverse cancers, including breast cancer [9, 10]. Cholesterol-biosynthetic enzymes have emerged as intriguing targets in many biological processes that support cancer stemness, tumor initiation, growth, and metastasis [11–20]. The genetic and pharmacological inhibition of cholesterol biosynthesis enzymes, such as 3-hydroxy-3-methylglutaryl-coenzyme A reductase (HMGCR), farnesyl-diphosphate farnesyltransferase 1 (FDFT1), farnesyl pyrophosphate synthase (FDPS), and 24-dehydrocholesterol reductase (DHCR24), have been shown to reduce cancer stem cell propagation, expression of stemness-related genes OCT4, SOX2, and NANOG, and tumorigenesis [18, 21–23]. NAD(P)-dependent steroid dehydrogenase-like (NSDHL) is an essential enzyme that catalyzes the NAD(P)+-dependent oxidative decarboxylation of the C4 methyl groups of 4-alpha-carboxysterols in post-squalene cholesterol biosynthesis [24]. Recently, NSDHL was identified as a regulator of oncogenesis in various cancer models [25–27]. We previously reported for the first time that NSDHL regulates breast cancer cell proliferation and migration abilities, anti-cancer drug sensitivity, and tumor growth and metastasis and that high NSDHL expression is associated with reduced relapse-free survival (RFS) in patients with ER + breast cancer [28]. However, little research has been conducted on the biological involvement of NSDHL in BCSCs maintenance and tumor-initiating capacity. We aimed to investigate whether NSDHL plays a role in maintaining BCSCs and to elucidate the molecular mechanism by which NSDHL regulates BCSCs in ER-positive MCF-7 tumor spheroids and orthotopic tumor xenograft models.

## Methods

### Cell lines and culture cell lines and culture

The ER + breast cancer cell lines (MCF-7 and ZR75-1) were used in this study. MCF-7 cells (ATCC^®^ HTB-22) were obtained from the American Type Culture Collection (Manassas, VA, USA) between 2012 and 2013. The ZR-75-1 cells (KCLB No. 21500) were obtained from the Korean Cell Line Bank (Seoul, Korea). MCF-7 cells were grown in DMEM (WelGENE, Seoul, Korea) supplemented with 10% fetal bovine serum (FBS) (WelGENE) and 1% penicillin-streptomycin (10,000 U/ml) (Gibco, Carlsbad, CA, USA). ZR-75-1 cells were grown in RPMI 1640 medium (WelGENE) supplemented with 10% fetal bovine serum (FBS) and 1% penicillin-streptomycin (Gibco). All the cells were maintained at 37 °C in a humidified atmosphere of 95% air and 5% CO2.

### Antibodies and drugs

For western blot, immunofluorescence staining, immunohistochemistry, and flow cytometry we used the following antibodies: β-actin (sc-47778), Vimentin (sc-6260), SOX2 (sc-365823), and NANOG (sc-293121) from Santa Cruz (CS, USA); NSDHL (ab190353), mCherry (ab167453), CD24 (ab202073), CD44 (ab6124), EpCAM (ab20160) and ALDH1A1 (ab227964) from Abcam (Cambridge, UK); Snail (#3879), PARP (46D11) (#9532), Cleaved PARP (Asp214) (#9544), Smad2 (#5339), Smad3 (#9523), Phospho-Smad2 (Ser465/467) (#3108) and Phospho-Smad3 (Ser423/425) (#9520) from Cell Signaling (Beverly, MA, USA); CD49f (NBP1-85747) from Novus Biologicals (Centennial, CO, USA); FITC-conjugated CD24 (555427), PE-conjugated CD44 Monoclonal Antibody (IM7) (550989), APC-conjugated CD44 Monoclonal Antibody (G44-26) (559942), and APC-conjugated EpCAM (347200) from BD Biosciences (Mansfield, MA, USA); FITC-conjugated CD49f (313606) from Biolegend (San Diego, CA, USA). Lovastatin (MK-803) (Selleckchem, Houston, TX, USA) was used for drug sensitivity testing of the HMG-CoA reductase (HMGCR) inhibitors. A-83-01 TGF-β RI kinase inhibitor (Selleckchem) was used to investigate the inhibitory effects of TGF-β signaling.

### siRNA transfection

NSDHL ON-TARGETplus SMART pool siRNA or ON-TARGETplus non-targeting siRNA pool was obtained from Dharmacon (Lafayette, CO, USA). The siRNA sequences are listed in Table S1. Briefly, 20 nM siRNA was diluted in Opti-MEM ^®^ I Reduced Serum Medium and mixed with Lipofectamine 2000 RNAiMAX Reagent (Thermo Fisher Scientific, Waltham, MA, USA). The mixture was then added to the cells and incubated for 48 h at 37 °C in a CO2 incubator.

### Short hairpin RNA lentiviral transduction

shRNA lentiviral particles (shNSDHL) (LPP-HSH103352-LVRU6MH-c-100) and control shRNA lentiviral particles (shCtrl) (LPP-CSHCTR001-LVRU6MH-025-C) were purchased from GeneCopoeia (Rockville, MD, USA). The short hairpin RNA (shRNA) sequences are listed in Table S1. Cells were seeded in each well of a 24-well plate 24 h before viral infection, and the medium was replaced with medium containing 5 µg/ml polybrene (sc-134220, Santa Cruz) and 2 × 10^6^ TU of lentiviral particles. After 72 h, the transduced cells were selected using 100 µg/ml hygromycin B Gold (InvivoGen, San Diego, CA, USA) for 7 days. Selected cells expressing mCherry were sorted using a FACSCalibur flow cytometer (BD Biosciences, Franklin Lakes, NJ, USA).

### Three-dimensional spheroid culture

Non-adherent three-dimensional culture, or “spheroid formation assay,” is widely used to assess the stemness potential of cancer cells. For spheroid formation, cells were seeded on an ultra-low attachment plate coated with polymer-X at a density of 2.5 × 10^5^ cells/ml DMEM (WelGENE) supplemented with 10% KnockOut™ Serum Replacement (Invitrogen, Carlsbad, CA, USA) and 1% penicillin-streptomycin (Gibco, Carlsbad, CA, USA). The culture medium was replaced every three days.

### Quantification of the spheroid size

Standalone image analysis software (ImageJ) was used to measure the spheroid size. To quantify the spheroid area, the processed image was loaded into ImageJ software and converted into an 8-bit image. After setting the scale to convert the pixel numbers to standard units, the red channel of the threshold was adjusted, and the resulting thresholded images were binary and only showed the spheroid region. After segmentation, the images were measured by providing the pixel size (0–infinity) and circularity (0.00–1.00). ImageJ software separately clustered the cells without background noise, and spheroid sizes were calculated for each spheroid.

### Cytotoxicity assay

To form 3D spheroids, the cells (1 × 10^5^/ml) were cultured in 96-well plates under the culture conditions described above. After 3 days, cells were treated with A-83-01 (0, 10, 50, 100µM), respectively, for 7 days. The cells were then determined using the CellTiter-Glo 3D Luminescent Cell Viability Assay (Promega, Madison, WI, USA), which measures the luminescence according to the adenosine triphosphate (ATP) in active cells. Equal amounts of the reagent were added to the cell culture medium, followed by shaking and stabilization. Luminescent signals were detected using a Varloskan Lux Multiplate Reader (Thermo Fisher Scientific).

### Quantitative reverse transcription-polymerase chain reaction (qRT-PCR)

Total RNA was extracted from the cells using the Tri-RNA reagent (FAVORGEN, Kaohsiung, Taiwan). RT-qPCR was performed using a cDNA kit (Takara; Kusatsu, Shiga, Japan). Real-time PCR was run on a Light Cycler 480 II (Roche, Salt Lake City, UT, USA) using SYBR Green PCR master mix (Applied Biosystems) and specific primers (Table S2). The results were analyzed using the ^Δ^CT method or 2^−ΔΔ^CT method, which reflects the threshold difference between a target gene and GAPDH in each sample, as well as the relative gene expression, with the reference sample set to 1 (control).

### RNA isolation and RNA-seq

Total RNA was extracted from BCC cells using TRIzol reagent (Invitrogen), according to the manufacturer’s instructions. The RNA concentration was measured using a NanoDrop 2000 spectrophotometer (Thermo Fisher Scientific). The RNA integrity number was determined using an Agilent RNA 6000 Nano Kit following the manufacturer’s protocol using an Agilent 2100 Bioanalyzer (Agilent, Santa Clara, CA, USA). Sequencing libraries were constructed using a QuantSeq 3’ mRNA-Seq Library Prep Kit (Lexogen, South Morang, Victoria, Australia) according to the manufacturer’s instructions. High-throughput RNA-seq was performed by single-end 75-bp sequencing using a NextSeq 500 system (Illumina, San Diego, CA, USA). Differentially expressed genes (DEGs) between BPA-exposed BCCs and control cells were determined based on counts from unique and multiple alignments using BEDtools coverage [29]. The read count data were processed based on the quantile normalization method using the EdgeR package in R using Bioconductor [30]. To select DEGs, we ranked genes with a *P* value < 0.05, using the log10 *P* value, and plotted them against the log2-fold change (FC) in a volcano plot. Upregulated and downregulated genes with *P* values < 0.05, and log2FC ratios > 0.59 were identified.

### Western blotting

The cells were lysed in RIPA buffer (Sigma, St. Louis, MO, USA). Proteins were separated using SDS-PAGE and transferred onto Immobilon–P transfer membranes (Merck Millipore, Bedford, MA, USA). After blocking with 5% non-fat dry milk in TBS-T or 5% BSA in TBS-T at room temperature for 1 h, the membranes were incubated with primary antibodies overnight at 4 °C and horseradish peroxidase-conjugated secondary antibodies at room temperature for 1 h and visualized using SuperSignal West Pico Chemiluminescent Substrate (Thermo Fisher Scientific) and Amersham Imager 600 (GE Healthcare, Buckinghamshire, UK). The relative intensities of the bands observed by western blotting were analyzed using ImageJ software (National Institutes of Health, Bethesda, MD, USA).

### Bio-plex Pro TGF-β immunoassays

TGF-β 1, 2, and 3 levels were determined using BioPlex Pro (TM) TGF-β Assay kits (Bio-Rad Laboratories, Inc., Hercules, CA, USA). The conditioned medium of spheroids cultured for 3 days was analyzed according to the manufacturer’s protocol. Fluorescence intensity was measured, and data analysis was performed using the Bio-Plex200 multiplex array system and Bio-Plex Manager 5.0 (Bio-Rad).

### Flow cytometry

The spheroids were digested with 0.25% trypsin, washed three times with PBS, resuspended in 1%BSA/PBS, stained with antibodies (CD44-PE, CD24-FITC, EpCAM-APC, and CD49f-FITC), or stained with their isotype controls at room temperature for 20 min. Flow cytometric analysis was performed on a BD FACSCanto II flow cytometer (BD Biosciences). The percentages of CD24+, CD44+, CD49f+, EpCAM+, CD44+/CD24-, and EpCAM+/CD49f + subpopulations were calculated using flow cytometry.

### Immunofluorescent staining

The spheroids were fixed with 4% paraformaldehyde, permeabilized in 1% BSA/PBS- Triton X-100 at 4 °C, and incubated with primary antibodies (NSDHL 1:10000, CD24, CD44 1:10, and EpCAM, CD49f 1:20) overnight at 4 °C and secondary antibodies (Alexa Fluor 488 goat anti-rabbit IgG (H + L) 1:1000) at room temperature for 15 min, followed by incubation with NucBlue^®^ Live REAdyProbes REAgent (Thermo Fisher Scientific). Images were acquired using a confocal microscope (Leica, Wetzlar, Germany).

### ALDEFLUOR assay

The ALDEFLUOR reagent system (Stem Cell Technologies, Vancouver, Canada) was used to determine the ALDH1 activity of the cells according to the manufacturer’s protocol. Cells (1 × 10^6^) dissociated from spheroids were suspended in ALDEFLUOR Assay buffer containing the ALDH substrate boron-dipyrromethene-aminoacetaldehyde (BAAA) and incubated for 40 min at 37 °C. The negative control was treated with a 50 mmol/L ALDH-specific inhibitor, diethylaminobenzaldehyde (DEAB). Cells with high ALDH activity were analyzed using a FACSCalibur flow cytometer (BD Biosciences).

### Total cholesterol assay

Total intracellular cholesterol levels in the spheroids were measured using a Total Cholesterol Assay Kit (Cell Biolabs, Inc., San Diego, CA, USA). Cholesterol standards and samples were prepared in accordance with the manufacturer’s instructions. A total of 25 µl of diluted cholesterol standards/serum samples and 25 µl of the cholesterol reaction reagent consisting of cholesterol esterase and cholesterol oxidase, a fluorometric probe, and horseradish peroxidase were added to each well. After incubation for 45 min at 37 °C, fluorescence signals were measured using a microplate reader (Synergy H1, BioTek Instruments, Inc., Winooski, VT, USA) at excitation and emission wavelengths of 550 and 595 nm, respectively. Cholesterol concentration in the samples was calculated by comparing the sample RFU with the cholesterol standard curve.

### Spheroid migration/invasion assay

After three days of spheroid formation, each spheroid was transferred to each well of a flat-tissue culture-treated 96-well plate for the spheroid migration assay. Spheroids were incubated in a culture medium containing 10% FBS at 37 °C. After 1 h of spheroid attachment, bright-field images of migrated spheroids were obtained every four hours. The migration distance was analyzed as the length compared with the previous time point. For the spheroid invasion assay, Matrigel at 4 °C was diluted with DMEM at a 1:3 ratio on ice, and 100 µl of it was added to 3-day-old spheroids in each well and incubated at 37 °C for 1 h. Next, 100 µl of 10% FBS DMEM was carefully added. Images of the spheroids were taken at 1-day intervals. The length of spheroid invasion was measured as the difference between the spheroid lengths at the previous time point. All data were analyzed using ImageJ software.

### Xenograft animal model

NOD.Cg-Prkdcscid Il2rg^tm1wjl^ /SzJ mice (NSG) were obtained from Jackson Laboratory (Bar Harbor, ME, USA). All animal experiments were approved by the Institutional Animal Care and Use Committee of Seoul National University (IACUC, SNU 200907-4) and performed in accordance with the ARRIVE guidelines. Forty female NSG mice were used in this study. Orthotopic xenografts were established by injecting 5 × 10^2^, 5 × 10^3^, 5 × 10^4^ and 1 × 10^6^ shCtrl- or shNSDHL spheroids mixed with 100 µl of Matrigel (BD Biosciences) into the fat pad of the fourth mammary gland of 5-week-old mice under anesthesia with 2–3% isoflurane (Ifran, Hana Pharm. Co., Ltd., Korea), and allowed to drink water supplemented with β-estradiol (6.25 µg/ml). After injecting tumor cells, the primary tumor volume was measured weekly using digital calipers and a modified ellipsoidal formula (volume = 1/2 [length×width^2^]). Eight weeks post-injection, mice were euthanized under 40% carbon dioxide inhalation, followed by cervical dislocation in accordance with the American Veterinary Medical Association guidelines for euthanasia of animals. Tumors and lungs were surgically removed for analysis. All experiments adhered to ARRIVE guidelines ( https://arriveguidelines.org).

### Immunohistochemistry

Primary tumors were fixed with 4% buffered paraformaldehyde, embedded in paraffin blocks, and sectioned into 4-µm thick sections. Sections were deparaffinized in xylene, rehydrated in a series of graded ethanol and water solutions, and pretreated by autoclaving at 98 °C for 20 min in citrate (pH 6.0) or 10 mM Tris/1 mM EDTA (pH 9.0) for antigen retrieval. Endogenous peroxidase activity was blocked by incubating with 3% H2O2 for 10 min at room temperature. After incubation with 10% normal goat serum for 1 h to block nonspecific binding of immunological reagents, primary antibodies were applied at 4 °C overnight, secondary antibodies were applied at room temperature for 60 min, and immunoreaction was visualized using a DAB chromogen kit (Agilent Technologies, Glostrup, Denmark). For double staining of ALDH1 and CD44, the DoubleStain IHC kit (ab183285) was used. Nuclei were counterstained with hematoxylin solution (Merck Millipore), according to the manufacturer’s instructions. Histological images of stained tissues were acquired using a microscope equipped with a CCD camera (Leica, Wetzlar, Germany).

### Survival outcomes analysis of the NSDHL mRNA expression levels and correlation analysis of NSDHL, NANOG, and SOX2 expression in patients with breast cancer

The relationship between NSDHL genes and relapse-free survival (RFS) in patients with breast cancer with a mean follow-up of 120 months was evaluated using Kaplan–Meier Plotter (http://www.kmplot.com/analysis) public microarray data repositories. The cutoff value for NSDHL expression was chosen as the median, which split the patient samples into two groups, and plots were generated accordingly. ER + or ER status was determined using immunohistochemistry and array analysis. The correlation between NSDHL, NANOG, and SOX2 expression in TCGA RNA-Seq results of patients with luminal-type breast cancer was evaluated using a Pairwise Spearman rank correlation test.

### Statistical analyses

For the analysis of data obtained in vitro and in vivo, graphs are presented as the mean ± standard deviation of at least three independent experiments. Statistical comparisons between two independent groups were performed using unpaired *t* tests. Data were analyzed using one-way analysis of variance (ANOVA), followed by the Tukey multiple comparison test for three or more groups. Statistical analyses were performed using GraphPad Prism v9.2.0 (GraphPad Software Inc., La Jolla, CA, USA). Statistical significance was defined as *P < 0.05, **P < 0.01, and ***P < 0.001.

## Results

### NSDHL knockdown repressed tumor sphere formation in MCF-7 and ZR-75-1 cells

**Fig. 1:**
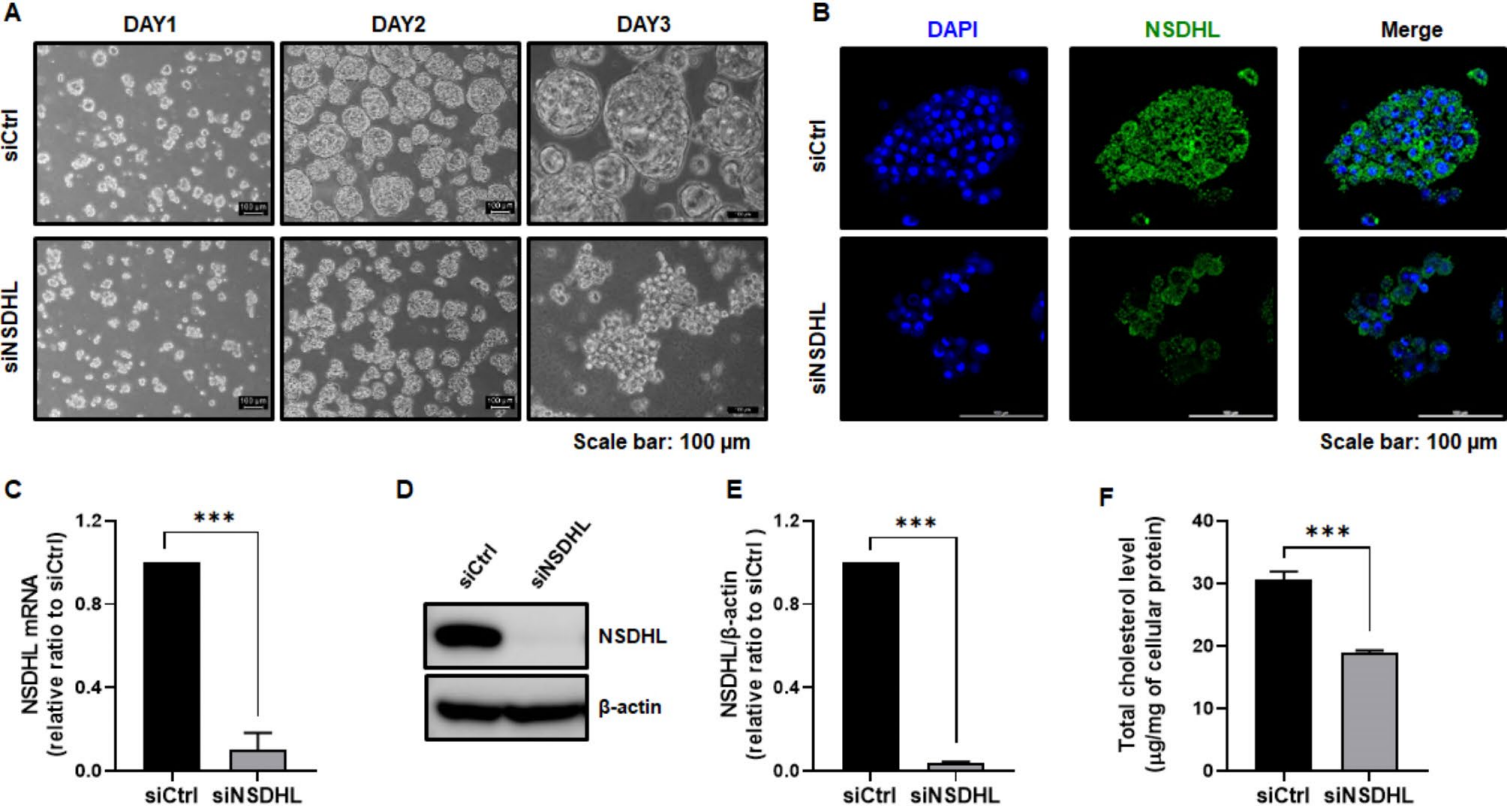
NSDHL knockdown suppresses spheroid formation and total cholesterol levels in the ER + breast cancer cell line MCF-7. **(A)** Formation of 3D-spheroids after 2–3 days of culturing control (siCtrl)- and NSDHL siRNA (siNSDHL) MCF-7 cells. **(B)** Representative images and immunofluorescence staining of DAPI (blue) and NSDHL (green) in control (siCtrl) and NSDHL siRNA (siNSDHL) MCF-7 spheroids. Scale bar: 100 μm. **C-E.** mRNA and protein levels in siNSDHL MCF-7 spheroids relative to siCtrl MCF-7 spheroids using qRT-PCR and western blot analysis (*n* = 6). **F.** Total cholesterol levels in siCtrl and siNSDHL MCF-7 spheroids (*n* = 9). All bar graphs represent mean ± standard deviation of at least three independent experiments. ***P < 0.001 as compared to siCtrl using unpaired t-tests

To investigate whether NSDHL affected tumor spheroid formation, siRNA was introduced into MCF-7 and ZR-75-1 cells. Compared with the controls, siNSDHL-MCF-7 spheroids were not tightly cohesive and formed round aggregates after suspension cultivation for three days (Fig. 1A). Immunofluorescence staining revealed a reduction in NSDHL expression in siNSDHL-MCF-7 spheroids compared to that in the control cells (Fig. 1B). Similarly, levels of NSDHL mRNA (0.10 ± 0.08, *P* < 0.001) and proteins (*n* = 6, 0.03 ± 0.01, *P* < 0.001) were significantly decreased in siNSDHL-MCF-7 spheroids compared to those in the controls (Fig. 1C-E). In ZR-75-1 cells, siRNA-mediated NSDHL knockdown significantly decreased spheroid size (*P* < 0.0001; Fig. S1 A) and NSDHL mRNA and protein levels in spheroids by 0.5-fold (*P* < 0.001, Fig. S1 B-C). NSDHL knockdown reduced the total cellular cholesterol levels from 30.55 ± 0.33 µg/mg to 18.84 ± 0.42 µg/mg (*n* = 3, *P* < 0.001) in MCF-7 spheroids cultured for 3 days (Fig. 1F).

### NSDHL knockdown decreased the number of BCSCs with CD44+/CD24- phenotype and high ALDH activity, as well as progenitors with the EpCAM+/CD49f + phenotype in MCF-7 spheroids

When compared to MCF-7 monolayers, MCF-7 spheroids upregulated the expressions of BCSC (CD44, ALDH1A1, ALDH1A2, and ALDH1A3)-, stemness (OCT4, SOX2, NANOG)-, or extracellular matrix (Col IV, FN-EDB, FN-EDA, and FN-IIICS)-related genes (Fig. S2A-B). The expression of genes encoding the cholesterol biosynthetic enzymes (HMGCR, PMVK, SQLE, LSS, NSDHL, and DHCR7) was considerably higher in MCF-7 spheroids than in monolayers (Fig. S2 C and G). Lovastatin, an HMGCR inhibitor, prevented spheroid formation in the MCF-7 cells (Fig. S3A). These findings imply a potential connection between the increased mRNA expression of cholesterol biosynthetic enzymes and the enrichment of BCSCs in MCF-7 spheroids.

As expected, NSDHL knockdown decreased the levels of HMGCR and DHCR7 and caused a significant decrease in the levels of BCSC (CD44)-, stemness (SOX2, NANOG)-, and extracellular matrix (FN-EDB)-related genes in MCF-7 spheroids (*n* = 6, Fig. S2D-F). These results show that NSDHL is not only involved in the expression of cholesterol biosynthetic enzymes but also in the expression of BCSC/stemness-related genes in MCF-7 spheroids.

**Fig. 2:**
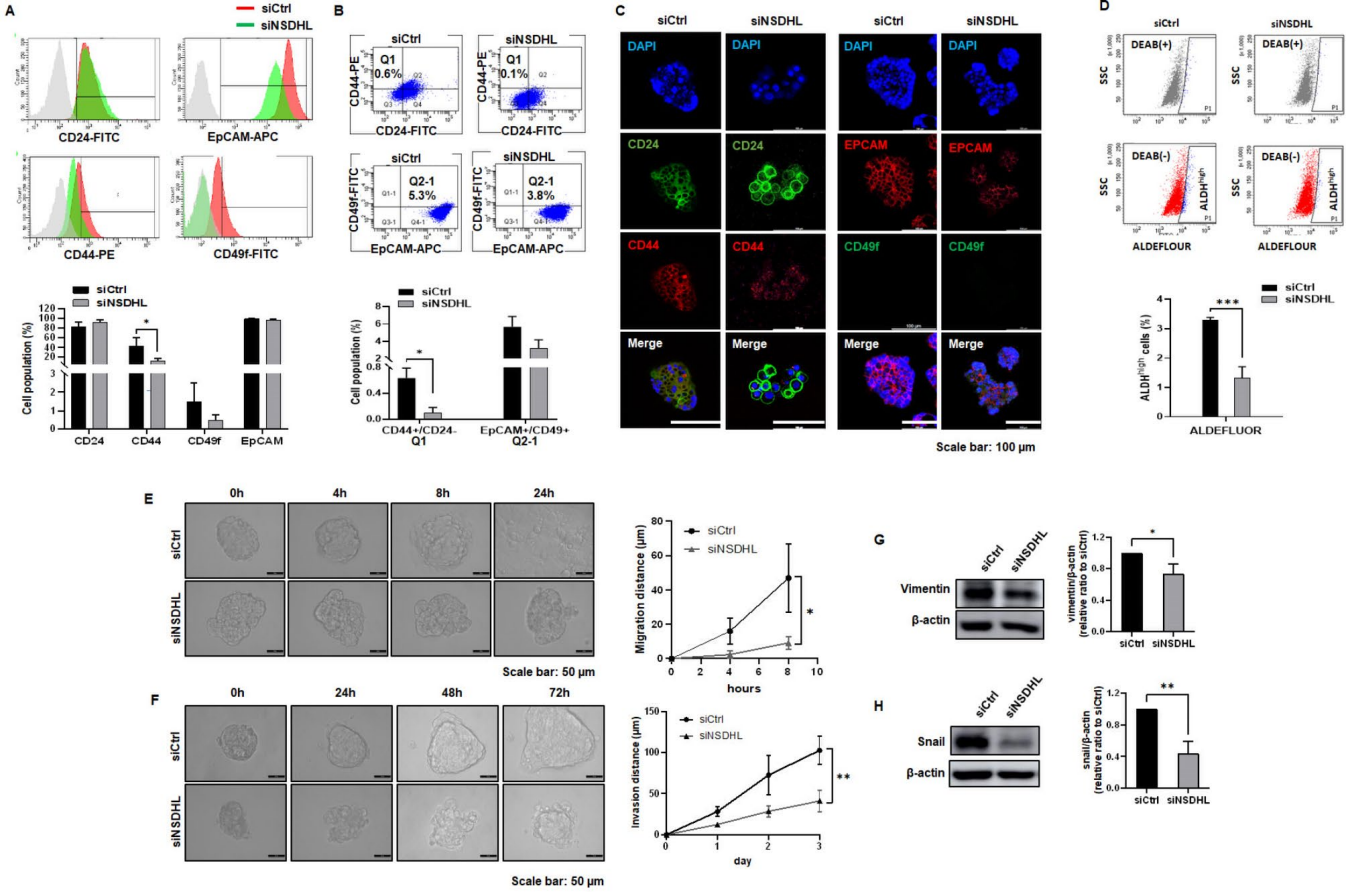
NSDHL knockdown reduced the number of BCSCs with CD44+/CD24 − phenotype and high ALDH activity in MCF-7 spheroids. **(A)** Representative flow cytometry histograms and quantification of CD44+, CD24+, CD49f+, or EpCAM + cells in siCtrl- and siNSDHL MCF-7 spheroids (*n* = 4). **(B)** Representative flow cytometry dot plots and quantification of CD44+/CD24- and EpCAM+/CD49f + cells in siCtrl and siNSDHL MCF-7 spheroids (*n* = 4). **(C)** Representative double immunofluorescence images of CD44+/CD24- and EpCAM+/CD49f- cells in siCtrl- and siNSDHL MCF-7 spheroids. CD24 and CD49f (green), CD44 and EpCAM (red), DAPI (blue) Scale bar: 100 μm. **(D)** Representative flow cytometry dot plots and quantification of ALDH + cells in siCtrl- and siNSDHL MCF-7 spheroids using ALDEFLUOR assay (*n* = 3). **E-F.** Representative images and quantification of the migrated distance from siCtrl- and siNSDHL-MCF-7 spheroid migration and invasion assays at various time points (*n* = 3). Scale bar: 50 μm. **G-H.** Representative western blot images and quantification data for Vimentin and Snail in siNSDHL-MCF-7 spheroids relative to siCtrl-MCF-7 spheroids (*n* = 3). Bar graphs show the mean ± standard deviation of at least three independent experiments. *P < 0.05, ***P < 0.001 as compared to siCtrl using unpaired t-tests

To investigate the impact of NSDHL knockdown on BCSC and progenitor subpopulations with CD44+/CD24- and EpCAM+/CD49f + phenotypes and high ALDH activity in MCF-7 spheroids, flow cytometry and immunofluorescence staining were performed. In comparison to controls, CD44 + cells (*n* = 3, from 42.8 ± 17.27 to 11.9 ± 6.24%, *P* = 0.043), EpCAM + cells (*n* = 3, from 99.5 ± 0.35% to 97.3 ± 1.05%, *P* = 0.100) and CD49f + cells (*n* = 3, from 1.5 ± 1.0% to 0.47 ± 0.31%, *P* = 0.16) decreased in siNSDHL-MCF-7 spheroids (Fig. 2A). CD44+/CD24- BCSCs (*n* = 3, from 0.63 ± 0.15% to 0.1 ± 0.08%, *P* = 0.0018) and EpCAM+/CD49f + progenitors (*n* = 3, from 5.67 ± 1.19% to 3.17 ± 1.01%, *P* = 0.05) decreased in siNSDHL-MCF-7 spheroids (Fig. 2B). Double immunofluorescence labeling revealed that siNSDHL-MCF-7 spheroids had fewer CD44+/CD24- and EpCAM+/CD49f + cells than controls (Fig. 2C). Similarly, BCSCs in siNSDHL-MCF-7 spheroids, identified by high levels of ALDH activity, were also reduced (*n* = 4, from 3.3 ± 0.08% to 1.33 ± 0.38%, *P* < 0.0001, Fig. 2D). In siNSDHL-ZR-75-1 spheroids, CD44 + cells decreased (*n* = 3, from 15.3 ± 0.7 to 8.77 ± 0.4%, *P* < 0.0001), while CD24 + cells increased (*n* = 3, from 23.83 ± 3.23 to 46.4 ± 3.38%, *P* = 0.0194), resulting in a decrease in CD44+/CD24- BCSCs (*n* = 3, from 5.9 ± 0.36 to 2.63 ± 0.06%, *P* = 0.0001) (Supplementary Fig. S4 A-D). Thus, NSDHL played a role in maintaining CD44+/CD24-BCSCs in MCF-7 and ZR75-1 spheroids.

BCSCs exhibit strong migratory and invasive capabilities. Spheroid migration and invasion assays were performed to determine whether NSDHL affects these features in MCF-7 spheroids. As shown in Fig. 2E, the migration distance in siNSDHL-MCF-7 spheroids was shorter than in controls (*n* = 3, after 4 h: 2.274 ± 2.36 μm vs. 15.97 ± 7.45 μm, *P* = 0.0386, after 8 h: 9.113 ± 3.63 μm vs. 46.92 ± 19.97 μm, *P* = 0.0321). Similarly, invasion distance in siNSDHL-MCF-7 spheroids was reduced compared to controls (*n* = 3, d1: 12.48 ± 1.50 μm vs. 28.13 ± 5.66 μm, *p* = 0.0098, d2: 28.45 ± 6.54 μm vs. 72.59 ± 24.35 μm, *P* = 0.0387, d3: 41.08 ± 12.98 μm vs. 102.7 ± 17.29 μm, *P* = 0.0079, Fig. 2F). In addition, the epithelial-mesenchymal transition markers vimentin (*n* = 3, 0.74 ± 0.12, *P* = 0.0207) and Snail (*n* = 3, 0.44 ± 0.15 m, *P* = 0.0027) were found to be lower in siNSDHL-MCF-7 spheroids compared to controls (Fig. 2G and H). These results imply that NSDHL stimulated EMT, which increased MCF-7 spheroid migration and invasion.

### RNA sequencing analysis revealed that NSDHL knockdown elicited widespread transcriptional changes in MCF-7 spheroids

**Fig. 3:**
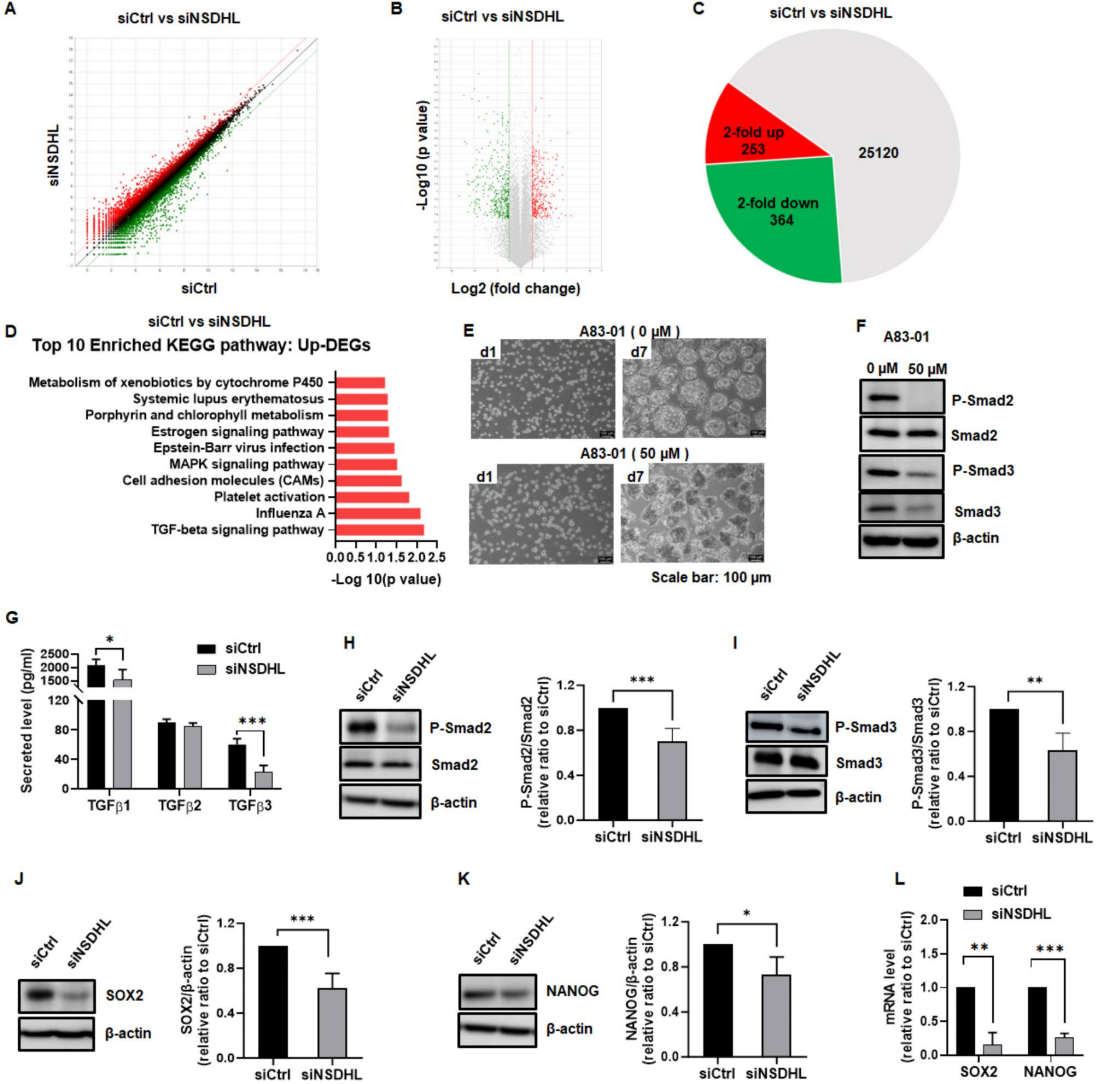
NSDHL knockdown led to a significant reduction in the secretion of TGF-β 1 and 3, phosphorylation of Smad2 and 3, and expression of SOX2 and NANOG in MCF-7 spheroids. **A-C.** Scatter plot, volcano plot, and Venn diagram of differentially expressed genes (DEGs) comparing NSDHL siRNA (siNSDHL)-and control siRNA (siCtrl)-transfected MCF-7 spheroids. **D.** Top 10 enriched KEGG pathway analyses of DEGs. **E-F.** Representative images of MCF-7 spheroids and western blotting images of phopho-Smad2 and 3, Smad2, and Smad3 in the presence or absence of A-83-01 (50 µM) for 7 days. Scale bar: 100 μm. **G.** Immunoassays of secreted TGF-β1, 2, and 3 levels measured from conditioned medium of siNSDHL- or siCtrl-spheroids (*n* = 6). **H-K.** Representative western blot images and quantification data of phopho-Smad2 and 3, Smad2, Smad3, SOX2, and NANOG in siNSDHL spheroids relative to siCtrl spheroids (*n* = 3). **L.** Relative mRNA levels of SOX2 and NANOG were assessed by qRT-PCR analysis in siNSDHL-spheroids and siCtrl-spheroids (*n* = 4). Bar graphs show the mean ± standard deviation of at least three independent experiments. *P < 0.05, ** *P* < 0.01, ***P < 0.001 as compared to monolayer using unpaired t-tests

To further explore the targets regulated by NSDHL, comprehensive gene expression profiles of NSDHL-knockdown MCF-7 and ZR-75-1 spheroids and controls were analyzed using RNA-Seq. Transcripts that were differentially regulated by log2 fold change ≥ 1 and *P* ≤ 0.05 are highlighted in red or green (Fig. 3A-B and Fig. S5 A-B). Comparison of siNSDHL-MCF-7 spheroids with controls showed 253-upregulation and 364-downregulation downregulated differentially expressed genes (DEGs) (Fig. 3C). However, the DEGs between siNSDHL-ZR-75-1 spheroids and controls were found by 32-upregulation and 90-downregulation (Fig. S5 C). In contrast to ZR-75-1 spheroids, NSDHL knockdown elicited widespread transcriptional changes in MCF-7 spheroids. Kyoto Encyclopedia of Genes and Genomes (KEGG) pathway analysis was performed to understand the biological functions and interactions of the genes modulated by NSDHL knockdown. The top 10 enriched KEGG pathways of the upregulated and downregulated DEGs are shown in Fig. 3D. The most enriched KEGG pathway term with the highest levels of gene representation was the TGF-β signaling pathway, suggesting that NSDHL knockdown may alter TGF-β signaling and lead to a change in the characteristics of MCF-7 spheroids, including stemness and tumorigenesis.

### NSDHL knockdown decreased the secretion of TGF-β1 and 3, phosphorylation of Smad2 and 3, and expression of SOX2 and NANOG in MCF-7 spheroids

Based on the KEGG pathway analysis of RNA-seq data, we further explored the molecular mechanism by which NSDHL regulates BSCSs. To assess whether blocking TGF-β-Smad signaling could affect tumor spheroid formation, we treated MCF-7 spheroids with the potent TGF-β type I receptor inhibitor A-83-01. Spheroids treated with A-83-01 (50 µM) appeared to be less cohesive without significant cytotoxicity and exhibited reduced phosphorylation of Smad 2 and Smad 3 (Fig. 3E-F and Fig. S6 A-C). A significant reduction in secreted levels of TGF-β1 (*n* = 6, 2088.97 ± 210.04pg/mL vs. 1542.46 ± 373.41pg/mL, *P* = 0.01) and TGF-β3 (*n* = 6, 60.13 ± 8.09 pg/mL vs. 23.3 ± 8.56pg/mL, *P* < 0.0001) was detected in siNSDHL-MCF-7 spheroids relative to control (Fig. 3G). The phosphorylation of Smad2 (*n* = 8, 0.70 ± 0.12, *P* < 0.0001) and Smad 3 (*n* = 6, 0.63 ± 0.15, *P* = 0.0002) was significantly decreased in siNSDHL-MCF-7 spheroids compared to the control (Fig. 3H-I).

According to RNA-seq data in MCF-7 spheroids, stemness-related genes, such as SOX2 and NANOG, which have been reported to regulate CSC properties in a wide range of cancers, were significantly decreased in siNSDHL compared to siCtrl (Table S3). In siNSDHL-MCF-7 spheroids, the protein levels of SOX2 (*n* = 7, 0.62 ± 0.13, *P* < 0.0001) and NANOG (*n* = 4, 0.73 ± 0.16, *P* = 0.0141) were significantly lower than in the control (Fig. 3J-K). Similarly, qRT-PCR analysis revealed that NSDHL knockdown significantly decreased the SOX2 and NANOG mRNA levels (Fig. 3L). These findings suggest that a reduction in the secreted levels of TGF-β1 and TGF-β3 by NSDHL knockdown results in a decrease in the phosphorylation of Smad2 and 3 and protein levels of SOX2 and NANOG, consequently leading to suppressed stemness of BCSC-enriched MCF-7 spheroids.

In addition, we examined whether the mechanism of BCSC regulation by NSDHL knockdown in ZR-75-1 spheroids was similar to that observed in MCF-7 spheroids. A-83-01 treatment decreased Smad 2/3 phosphorylation in ZR-75-1 spheroids (Fig. S5 D). As shown in Fig. S5 E-H, siNSDHL-ZR-75-1 spheroids displayed a significant decrease in the phosphorylation of SMAD3 (*n* = 3, 0.20 ± 0.21, *P* = 0.0028), but not SMAD2 (*n* = 3, 0.90 ± 0.20, *P* = 0.44). SOX2 expression did not significantly decrease (*n* = 3, 0.64 ± 0.35, *P* = 0.1506) and NANOG expression slightly increased (*n* = 3, 1.21 ± 0.44, *P* = 0.4571) in siNSDHL-ZR-75-1 spheroids compared to controls (Fig. S5. I-L). Along with the aforementioned RNA-seq results for ZR-75-1 spheroids, our findings indicate that NSDHL may regulate BCSC-enriched spheroids through different mechanisms, even within ER + breast cancer cells.

### NSDHL knockdown represses the tumorigenic potential of MCF-7 spheroids in a xenograft mouse model

**Fig. 4:**
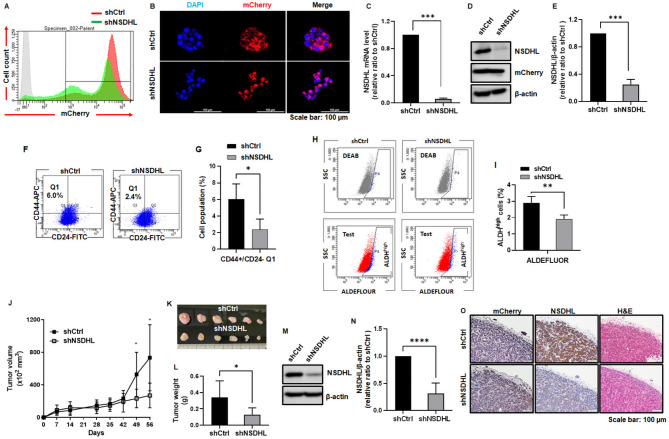
NSDHL knockdown suppresses tumor initiation and growth in tumor spheroid-injected xenograft tumor models. **(A)** Representative flow cytometry histogram of mCherry-positive cells sorted from NSDHL shRNA (shNSDHL) or control shRNA (shCtrl)-tagged mCherry-transduced MCF-7 cells. **(B)** Representative confocal images of mCherry-positive cells in shNSDHL- or shCtrl-spheroids. mCherry (red) and DAPI (blue). Scale bar: 100 μm **C-E.** NSDHL mRNA and protein levels and representative western blot images of NSDHL and mCherry in shNSDHL-MCF-7 spheroids relative to shCtrl-MCF-7 spheroids by qRT-PCR (*n* = 6) and western blot analysis (*n* = 6). **F-G.** Representative flow cytometry dot plots and quantification of CD44+/CD24 − cells in shCtrl and shNSDHL MCF-7 spheroids (*n* = 3). **H-I.** Representative flow cytometry dot plots and quantification of ALDH + cells in shCtrl- and shNSDHL-MCF-7 spheroids using ALDEFLUOR assay (*n* = 3). **J.** Tumor volume was measured weekly in shCtrl- or shNSDHL-MCF-7 spheroid-injected mice (*n* = 6) for 56 days post-injection. **K-L.** Gross images and wet weights of tumors removed from shNSDHL- or shCtrl-MCF-7 spheroid-injected mice (*n* = 6) at 56 days post-injection. **M-N.** Representative western blot images and quantification data of NSDHL proteins in shNSDHL-MCF-7 tumors compared to shCtrl-MCF-7 tumors (*n* = 6). **O.** Representative NSDHL and mCherry immunohistochemistry images and H&E images of shNSDHL- or shCtrl-MCF-7 tumor tissues. Scale bar: 100 μm. The bar graph shows the mean ± standard deviation. *P < 0.05, ** *P* < 0.01, ***P < 0.001 as compared to shCtrl using unpaired t-tests

To address the effects of NSDHL knockdown on tumorigenicity in xenograft mouse models, MCF-7 cells were transfected with mCherry-tagged NSDHL or control shRNA. mCherry-positive MCF-7 cells stably expressing shCtrl or shNSDHL were sorted 99% (Fig. 4A-B). NSDHL shRNA transduction effectively reduced NSDHL mRNA and protein levels from 1 to 0.06 ± 0.01 and 0.25 ± 0.07 respectively in MCF-7 spheroids (*n* = 6, *P* < 0.0001, Fig. 4C-E). CD44+/CD24- BCSCs (*n* = 3, from 6.03 ± 1.52% to 2.37 ± 1.03%, *P* = 0.0476) were decreased in shNSDHL-MCF-7 spheroids relative to controls (Fig. 4F-G). BCSCs with high ALDH activity were also reduced in shNSDHL-MCF-7 spheroids (*n* = 3, from 2.90 ± 0.40% to 1.90 ± 0.26%, *P* = 0.022, Fig. 4H-I).

To evaluate the impact of stable NSDHL knockdown on the tumorigenic potential of MCF-7 spheroids, various numbers of cells from shCtrl and shNSDHL MCF-7 spheroids were injected into the mammary fat pads of the mice. As shown in Table 1, shCtrl-MCF-7 spheroids induced tumor formation within 30 days, even when as few as 500 cells per mouse were injected, whereas shNSDHL-MCF-7 spheroids failed to induce tumor formation when < 500 cells were injected. After injection with 1 × 10^6^ cells of shCtrl and shNSDHL MCF-7 spheroids, NSDHL knockdown resulted in a significant reduction in tumor volume (shCtrl vs. shNSDHL; *n* = 6, 527.79 ± 270.95 vs. 232.09 ± 113.86 mm^3^ at 49 days, *P* = 0.033 and 733.06 ± 405.53 vs. 270.04 ± 152.20 mm3 at 56 days, *P* = 0.026, respectively) and tumor weights (shCtrl vs. shNSDHL; *n* = 6, 0.34 ± 0.21 g vs. 0.13 ± 0.08 g at 56 days, *P* = 0.043) (Fig. 4J-L). In shNSDHL tumors, a significant decrease in NSDHL protein levels (shNSDHL; *n* = 6, 0.31 ± 0.19, *P* < 0.0001, Fig. 4M-N) and low staining intensity of NSDHL were observed (Fig. 4O). These results suggest that NSDHL is responsible for tumor initiation and growth of MCF-7 spheroids.

**Table 1:** Incidence of palpable tumors in mice injected with shCtrl or shNSDHL cells

Cell dose	Palpable tumor (%)		
	shCtrl group	shNSDHL group	Termination (day)
5 × 10^2^	60% (3/5)	0% (0/5)	30
5 × 10^3^	80% (4/5)	20% (1/5)	30
5 × 10^4^	100% (5/5)	60% (3/5)	30
1 × 10^6^	100% (6/6)	83% (5/6)	30

### NSDHL knockdown caused a reduction in BCSCs with the CD24-/CD44 + phenotype and high ALDH activity in MCF-7 tumor tissues, accompanied by a significant decrease in Smad2/3 phosphorylation

**Fig. 5:**
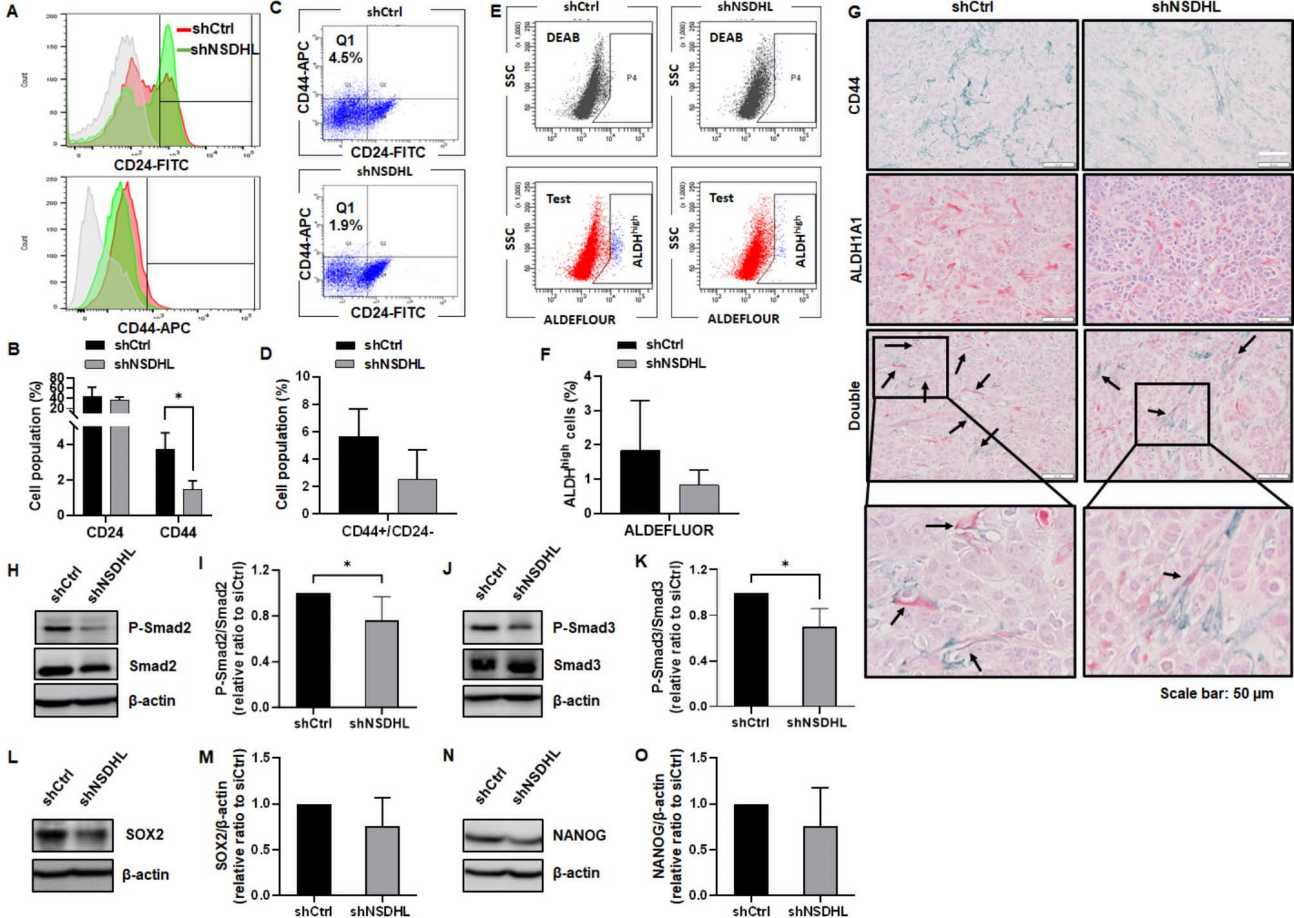
NSDHL knockdown reduced the BCSC population with CD44+/CD24 − phenotype and ALDH activity in tumor spheroid-injected xenograft tumor tissues, accompanied by decreased Smad2/3 phosphorylation and SOX2 expression. **A-D.** Representative flow cytometry histograms, dot plots, and quantification of CD44 + and CD24- cells in control (shCtrl) and NSDHL shRNA (shNSDHL)-transduced MCF-7 tumors (*n* = 4). **E-F.** Representative flow cytometry dot plots and quantification of ALDH + cells in shCtrl- and shNSDHL MCF-7 tumors using ALDEFLUOR assay (*n* = 4). **G.** Representative double immunohistochemistry images of CD44 + and ALDH1A1 + cells in shCtrl and shNSDHL MCF-7 tumors. CD44 (green), ALDH1A1(red). Double CD44+/ALDH1A1 + cells (arrow). **H-O.** Representative western blot images and quantification data of phopho-Smad2 and 3, Smad2, Smad3, SOX2, and NANOG levels in shNSDHL MCF-7 tumors relative to shCtrl MCF-7 tumors (*n* = 5). All graphs show the mean ± standard deviation. *P < 0.05, compared with shCtrl using unpaired t-tests

BSCS subpopulations with the CD44+/CD24- phenotype or high ALDH activity were analyzed to verify the role of NSDHL in maintaining the BCSC population within tumor tissues. The population of CD44 + cells in shNSDHL tumors (1.48 ± 0.47%) was reduced compared to controls (3.70 ± 0.96) (*n* = 4, *P* = 0.006, Fig. 5A-B), whereas the population of CD24 cells did not change substantially. CD44+/CD24- BCSCs decreased in shNSDHL tumors (2.5 ± 2.15%) relative to controls (5.68 ± 1.98%) (*n* = 4, *P* = 0.073, Fig. 5C-D). BCSCs with high ALDH activity in tumors, as assessed by the ALDEFLUOR assay, were also reduced in shNSDHL tumors (0.83 ± 0.44%) relative to controls (1.83 ± 1.46%) (*n* = 4, *P* = 0.237, Fig. 5E-F). Double immunohistochemical staining showed a reduction in the number of CD44+/ALDH + cells in shNSDHL tumor tissues (Fig. 5G). The levels of phosphorylated Smad2 (*n* = 5, 0.76 ± 0.21, *P* < 0.032) and phosphorylated Smad3 (*n* = 5, 0.70 ± 0.16, *P* = 0.003) were significantly reduced in shNSDHL tumors relative to controls (Fig. 5H-K). Protein levels of SOX2 (*n* = 5, 0.76 ± 0.30, *P* = 0.12) and NANOG (*n* = 4, 0.76 ± 0.0.42, *P* = 0.298) were reduced in shNSDHL tumors relative to controls (Fig. 5L-O). These results show that NSDHL contributes to the maintenance of BCSCs and regulation of the TGF-β/Smad signaling cascade in MCF-7 tumor models.

### A positive correlation between the expression of NSDHL and SOX2 was found in patients with luminal breast cancer

**Fig. 6:**
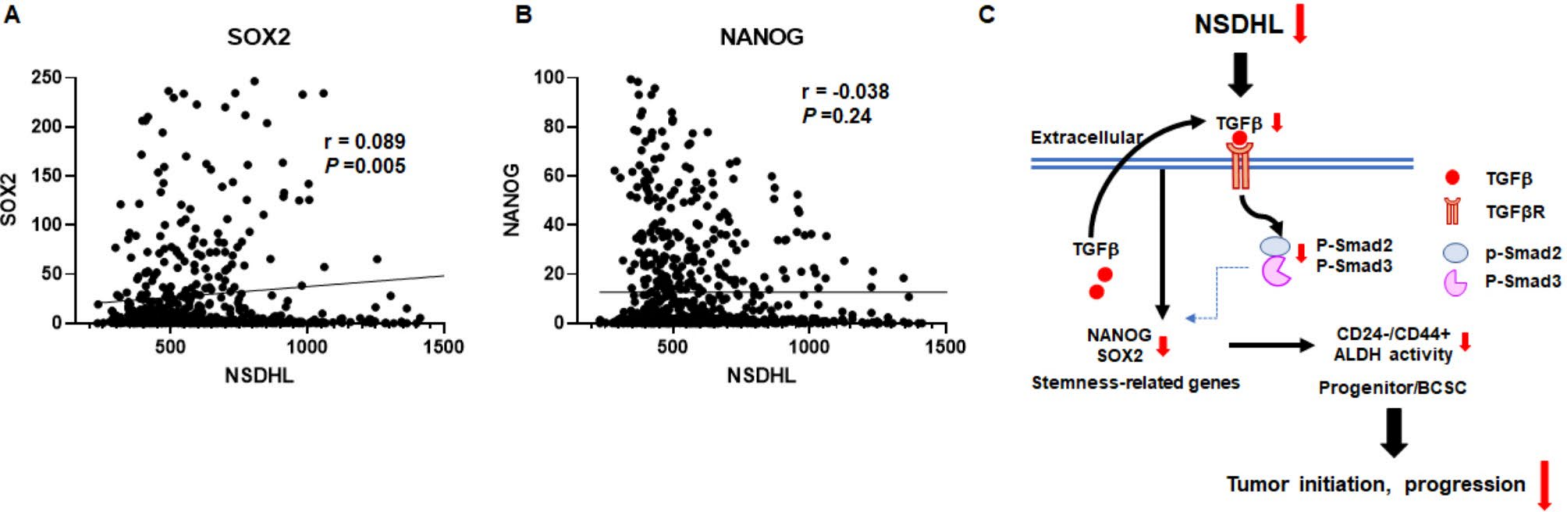
A positive correlation was observed between NSDHL and SOX2 gene expression. **A-B.** Analysis of correlation among NSDHL, SOX2, and NANOG gene expression obtained from RNA-seq in tumor tissues of patients with luminal type breast cancer based on TCGA database (*N* = 998) according to the Spearman rank correlation. **C.** Schematic diagram of the regulatory mechanisms of NSDHL knockdown in maintaining the BCSC population and tumor-initiating capacity in ER + breast cancer

To determine whether there were any correlations between NSDHL and BCSC-related genes in specimens of patients with luminal-type breast cancer, we analyzed the expression levels of NSDHL and BCSC-related genes SOX2 and NANOG obtained from RNA-seq of breast tumor tissues (*N* = 998) based on the TCGA database. The Spearman correlation analysis was used to evaluate the relationships among NSDHL, SOX2, and NANOG levels. Analysis based on the Spearman correlation coefficient indicated a very weak positive correlation between NSDHL and SOX2 expression in patient tumor tissues (*n* = 998, *r* = 0.089, *P* = 0.005; Fig. 6A), whereas a negative correlation was found between NSDHL and NANOG expression (*n* = 998, *r* = -0.038, *P* = 0.24; Fig. 6B). These findings highlight the co-regulated or synchronized biological functions of NSDHL and SOX2 in sustaining BCSCs in breast cancer.

### High NSDHL expression is associated with recurrence in patients with ER + breast cancer

In the Kaplan–Meier Plotter database, patients with ER + breast cancer with high NSDHL expression had a lower 10-year RFS rate (Probe ID 209279_s: *n* = 2561, HR = 1.22, 95% CI = 1.04–1.43, *P* = 0.014; Probe ID 215093: *n* = 2561, HR = 1.34, 95% CI = 1.14–1.57, *P* = 0.00027) compared to those with low NSDHL expression (Fig. S7). These findings imply that NSDHL may be associated with ER status and may contribute to an increased risk of relapse in ER + breast cancer cells. In this study, we investigated the involvement of NSDHL in the regulation of BCSCs, which are thought to be responsible for ER + breast cancer recurrence.

## Discussion

Accumulated reports have shown that cholesterol and rate-limiting enzymes of cholesterol biosynthesis are crucial for the maintenance and propagation of cancer stem cells in diverse cancers, including breast cancer [16–18, 20, 22, 31, 32]. In a previous study, we first reported NSDHL as a biomarker of poor prognosis to accelerate breast tumor growth and metastasis and to reduce RFS in patients with ER + breast cancer [28]. In this study, we hypothesized that NSDHL, owing to its significance in ER + breast cancer, is involved in the maintenance and propagation of BCSCs. The siRNA/shRNA-mediated knockdown of the NSDHL gene inhibited spheroid formation of ER + breast cancer cells grown on ultra-low attachment plates and tumorigenesis in an orthotopic breast cancer mouse model, leading to a reduction in BCSCs/progenitors with CD44+/ CD24- and EpCAM+/CD49f + phenotypes, and high ALDH activity in MCF-7 spheroids and xenograft tumors. Mechanistically, NSDHL knockdown impaired the cancer stemness characteristics of BCSCs by suppressing TGF-β signaling, which in turn decreased the secretion of TGF-β 1 and TGF-β3, inactivated Smad2 and Smad3, and downregulated the expression of SOX2 and NANOG, key regulators of cancer stemness (Fig. 6C).

Depletion of HMGCR and FDPS genes and drugs (lovastatin, alendronate/zoledronate, and squalestatin 1) targeting specific HMGCR, FDPS, and FDFT1 enzymes impairs spheroid formation in colon cancer and glioblastoma [19, 21], implying an important role for cholesterol biosynthetic enzyme genes in stemness and tumorigenesis. In the present study, lovastatin, a HMCGR inhibitor, suppressed spheroid formation in MCF-7 cells. As hypothesized, NSDHL knockdown inhibited spheroid formation in MCF-7 cells grown on polymer-X-coated ultra-low attachment plates and delayed tumor initiation in a mouse tumor model by injecting MCF-7 spheroids. These findings suggest that ER + breast cancer cells rely on NSDHL to form BCSC-enriched spheroids and support tumor initiation, thereby promoting breast tumor development and progression.

The most commonly used markers of BCSCs are CD44+, CD24−/low, CD49f+, and EpCAM+ [1]. Most BCSCs with the CD44+/CD24-/low phenotype exist as luminal progenitors with the EpCAM+/CD49f + phenotype [33]. Another BCSC was identified based on its high ALDH activity [34]. We found that NSDHL knockdown in MCF-7 cells reduced the number of BCSCs with CD44+/ CD24 − phenotype and high ALDH activity and progenitors with the EpCAM+/CD49f + phenotype in an ultra-low attachment in vitro culture system and xenograft tumors, thus substantiating NSDHL’s critical role of NSDHL in BCSC maintenance.

Stemness, known as the self-renewal capacity of cancer stem cells, is regulated by many signaling pathways, including TGF-β, Hedgehog, Wnt, Notch, and FGF, and transcription factors, including NANOG, OCT4, SOX2, KLF4, and c-MYC [35]. In the present study, KEGG pathway analysis based on RNA-seq revealed that the TGF-β signaling pathway was enriched in NSDHL-knockdown MCF-7 spheroids, implying a link between NSDHL and TGF-β signaling pathways in the regulation of BCSCs. We observed a decrease in the secretion of TGF-β1 and TGF-β3 in NSDHL-knockdown MCF-7 spheroids relative to that in the control, suggesting that NSDHL may be involved in TGF-β secretion. Further studies are required to explore the mechanisms by which NSDHL regulates TGF-β expression and secretion.

A decrease in TGF-β-mediated collagen fibril and fibronectin production can inhibit spheroid-forming efficiency [36, 37]. TGF-β induces the self-renewal capacity of BCSCs and promotes BCSC migration by inducing EMT [15, 38, 39]. Concurrently, we found a decrease in TGF-β1 and TGF-β3 with a simultaneous reduction in the mRNA expression of collagen I/IV, fibronectin splicing variant EDB-FN fibronectin, vimentin, and snail, consequently impairing spheroid formation and migratory abilities in NSDHL-knockdown MCF-7 spheroids relative to the control.

Recently, evidence has emerged for a connection between the genetic and pharmacological inhibition of cholesterol biosynthesis and TGF-β-mediated signaling pathways in the context of cancer stem cell maintenance and cancer progression to metastasis [19, 28, 36]. Treatment with atorvastatin or NSDHL knockout activates SREBP1, which promotes TGF-β1 expression and induces a canonical Smad2 and Smad3 effector cascade, resulting in the induction of epithelial-mesenchymal transition in pancreatic cancer [16]. Contrary to the findings on NSDHL-knockdown pancreatic cancer cells, Chen et al. reported that NSDHL knockdown impairs the TGF-β-mediated Smad3 signaling pathway by inducing the endosomal degradation of TGF-β receptor 2 to serve as a promoter of cancer cell proliferation and metastasis in breast cancer cells (MDA-MB-231) [26], which supports our current findings. In light of the aforementioned studies, the TGF-β-mediated molecular mechanisms by which NSDHL regulates the biological activities of cancer cells may differ among cancer types.

The molecular mechanisms linking NSDHL- and TGF-β-mediated signaling to the enrichment and maintenance of BCSCs remain poorly understood. This study revealed that NSDHL knockdown leads to a reduction in the secretion of TGF-β1 and TGF-β3, as well as the phosphorylation of Smad2 and Smad3, which are part of the TGF-β signaling cascade in MCF-7 spheroids and xenograft tumors. We also showed that the stemness-related transcription factors SOX2 and NANOG, in the context of the TGF-β downstream signaling cascade, are linked to NSDHL, which is consistent with TGF-β-mediated signaling to enrich and maintain the stemness of breast cancer cells [40, 41].

BCSCs have also been suggested as the basis of cancer relapse following initial therapy [42, 43]. In the present study, high NSDHL expression was associated with shorter RFS in patients with ER + breast cancer. Additionally, based on RNA-Seq from the TCGA database, we revealed a significant positive correlation between NSDHL and SOX2 in the luminal breast tumor tissues of patients. Further studies are needed to investigate the genetic networks between NSDHL and other BCSC-related genes to deepen our understanding of the role of NSDHL in regulating BCSC phenotypes.

## Conclusions

In conclusion, our findings indicate that the NSDHL gene contributes to spheroid formation of ER + human breast cancer cell lines MCF-7 and ZR75-1 and maintains BCSCs/progenitors with CD44+/CD24- and EpCAM+/CD49f + phenotypes and high ALDH activity by regulating TGF-β signaling and key regulators of cancer stemness, SOX2 and NANOG, in MCF-7 spheroids and xenograft tumors.

## Electronic supplementary material

Below is the link to the electronic supplementary material.

**Supplementary Material 1**: **Figure S1.** Analysis of spheroid formation capacity and NSDHL expression in ER + breast cancer cell line ZR-75-1 spheroids. A. Representative images and quantification of the spheroid size of siCtrl- or siNSDHL-transfected ZR-75-1 cells grown on ultralow-attachment plates coated with Polymer-X. Scale bar: 100 µm. B-C.–C Relative expression levels of NSDHL mRNA and protein in ZR-75-1 spheroids transfected with control (siCtrl) or NSDHL siRNA (siNSDHL). The bar graphs show the mean ± standard deviation of six independent experiments. ***P < 0.001 and ****P < 0.0001 as compared to siCtrl using unpaired t-tests.

**Supplementary Material 2**: **Figure S2.** Analysis of the expression of BCSC- and cholesterol-related genes in MCF-7 spheroids compared to that in monolayers. A-C. Relative mRNA levels of breast cancer stem cell-related genes (CD24, CD44, ALDH1A1, ALDH1A2, ALDH1A3, OCT4, SOX2, and NANOG), extracellular matrix-related genes (COLI, COLIV, FN-EDB, FN-EDA, and FN-IIICS), and cholesterol biosynthetic enzymes (HMGCR, PMVK, SQLE, LSS, CYP51A1, NSDHL, and DHCR7) by qRT-PCR analysis in monolayer and spheroids of MCF-7 cells cultivated for 3 days. D-F. The relative mRNA levels of cholesterol biosynthetic enzymes, breast cancer stem cell-related genes, and extracellular-matrix-related genes were assessed by qRT-PCR analysis in siCtrl or siNSDHL-MCF-7 spheroids cultivated for 3 days. G. Diagram of cholesterol biosynthetic pathway and enzymes. The bar graphs show the mean ± standard deviation of six independent experiments. *P < 0.05, **P < 0.01, ***P < 0.001 as compared to siCtrl using unpaired t-tests.

**Supplementary Material 3**: **Figure S3.** Lovastatin, an HMGCR inhibitor, strongly suppressed spheroid formation in MCF-7 cells. A. Representative images of spheroids of MCF-7 cells grown on ultra-low attachment plates in the presence or absence of lovastatin (2.5 µM) for three days.

**Supplementary Material 4**: **Figure S4.** Impact of NSDHL knockdown on BCSCs with CD44+/CD24 − phenotype in ZR-75-1 spheroids. A-D. Representative flow cytometry dot plots and quantification of CD44+, CD24 −, and CD44+/CD24 − cells in control (siCtrl) and NSDHL siRNA (siNSDHL)-transfected ZR-75-1 spheroids. The data represent the mean ± standard deviation (SD) of three independent experiments. *P < 0.05, ***P < 0.001, ****P < 0.0001 as compared to siCtrl using paired t-tests.

**Supplementary Material 5**: **Figure S5.** RNA-seq analysis and the expression of phosphorylation of SMAD 2/3, SOX2, and NANOG in ZR-75-1 spheroids. A-C. Scatter plot, volcano plot, and Venn diagram of differentially expressed genes (DEGs) between siNSDHL-transfected and siCtrl-transfected ZR-75-1 spheroids. D. Representative western blot images of phopho-Smad2/3 and Smad2/3 in ZR-75-1 spheroids cultivated in the presence or absence of A-83-01 (50 µM) for seven days. E-L. Representative western blot images and quantification of the relative expression levels of NSDHL, phopho-Smad2/3, Smad2/3, SOX2, and NANOG in siCtrl and siNSDH spheroids of ZR75-1 cells cultivated for 3 days. Bar graphs show the mean ± standard deviation of three or four independent experiments. **P < 0.01 as compared to siCtrl using unpaired t-tests.

**Supplementary Material 6**: **Figure S6.** A-83-01 (50 µM) decreased the phosphorylation of both SMAD2 and SMAD3 but did not show significant toxicity in MCF-7-spheroids. A. Representative western blot images of phosphorylation-Smad2/3 and Smad2/3 in MCF-7 spheroids at 0, 10, and 50 µM A-83-01. B. The percentage cell viability was assessed using the CellTiter-Glo® 3D luminescent cell viability assay in MCF-7 spheroids treated with A-83-01. C. Representative western blot images and quantification data of the relative expression levels of PARP-1 and Cleaved-PARP-1 in MCF-7 spheroids after A-83-01 treatment (0, 10, 50, and 100 µM). MCF-7 spheroids were cultivated with 0, 10, 50, and 100 µM A-83-01 for 7 days. The data represent the mean ± standard deviation of three independent experiments.

**Supplementary Material 7**: **Figure S7.** High NSDHL expression is associated with shorter relapse-free survival (RFS) in patients with ER + breast cancer. A. KM plotter analysis of NSDHL (probe ID: 20927_S and 215093) gene expression and RFS in patients with ER + breast cancer.

**Supplementary Material 8**: **Table S1.** Sequences of siRNAs and shRNAs. **Table S2.** Specific primer sequences used for real-time RT-PCR. **Table S3.** RNA-Seq analysis of siCtrl- or siNSDHL MCF-7-spheroids.

## Data Availability

The datasets generated and/or analyzed during the current study are available in the Genome Sequence Archive (GSA) repository (https://ngdc.cncb.ac.cn/gsa/) with GSA-Human submission ID (subHRA011958) and the BioProject accession number (PRJCA029069). And all other data generated and/or analyzed during this study are included in this published article and its supplementary information files.

## Abbreviations

NSDHL: NAD(P)-dependent steroid dehydrogenase-like
BCSCs: Breast cancer stem-like cells
ER+: Estrogen receptor-positive
HMGCR: 3-hydroxy-3-methylglutaryl-coenzyme A reductase
FDFT1: Farnesyl-diphosphate farnesyltransferase 1
FDPS: Farnesyl pyrophosphate synthase
DHCR24: 24-dehydrocholesterol reductase
RFS: Relapse-free survival
qRT-PCR: Quantitative reverse transcription-polymerase chain reaction
DEGs: Differentially expressed genes
KEGG: Kyoto Encyclopedia of Genes and Genomes
