# Supplementary material for: NSDHL contributes to breast cancer stem-like cell maintenance and tumor-initiating capacity through TGF-β/Smad signaling pathway in MCF-7 tumor spheroid
Source: BMC Cancer. 2024 Nov 8;24:1370. doi: 10.1186/s12885-024-13143-3 (PMC11549796; doi:10.1186/s12885-024-13143-3)
Supplement: Supplementary file 8 — Supplementary Material 8: Table S1. Sequences of siRNAs and shRNAs. Table S2. Specific primer sequences used for real-time RT-PCR. Table S3. RNA-Seq analysis of siCtrl- or siNSDHL MCF-7-spheroids. [file 12885_2024_13143_MOESM8_ESM.docx]

**Table S1. Sequences of siRNAs and shRNAs.**

|  | **Target Sequence** | |
| --- | --- | --- |
| NSDHL siRNA pool | #1 | GAGGAUAUGCUGUCAAUGU |
|  | #2 | GCAGUUAGCGAGCCAAUGA |
|  | #3 | GCCAAGAGAUGCACAGUGA |
|  | #4 | GGGAAGGCAUUUCACAUCA |
| Nontargeting siRNA pool | #1 | UGGUUUACAUGUCGACUAA |
|  | #2 | UGGUUUACAUGUUGUGUGA |
|  | #3 | UGGUUUACAUGUUUUCUGA |
|  | #4 | UGGUUUACAUGUUUUCCUA |
|  | #1 | GCGTCGATATCAAGAATGGAA |
| NSDHL shRNA | #2 | CCAACGATCCTGAGAAGAATT |
|  | #3 | GGCAAGATGAAGTTCGTGATT |
| Control shRNA | #1 | TGCGTACACCTTAATCACC |

**Table S2. Specific primer sequences used for real-time RT-PCR.**

| **Gene** | **Sequence (5′**$\boldsymbol{->}$**3′)** | |
| --- | --- | --- |
| NSDHL | Forward | GGTGACGCACAGTGGAAAAC |
|  | Reverse | TCGCACGGACTCATTTGACA |
| HMGCR | Forward | GGGAACCTCGGCCTAATGAA |
|  | Reverse | CACCACGCTCATGAGTTTCCA |
| PMVK | Forward | GCCTTTCTCTCCGCGTGTCT |
|  | Reverse | GGAGCGGCAACAAGGAACA |
| SQLE | Forward | GCGTGCTTGGCTCTGCTTT |
|  | Reverse | CCTGGGCATCAAGACCTTCCA |
| LSS | Forward | GCACTGGACGGGTGATTATGGT |
|  | Reverse | CGCAGGTACCGCACAATCTCTT |
| CYP51A1 | Forward | CAGGGATTGATCCGCCTCTTCA |
|  | Reverse | CACAGAATGGGGCGGGATGTT |
| DHCR7 | Forward | GGGGCCGGTTCAAGAAGGAAA |
|  | Reverse | GCCCTTGAGATGCGGTTCTGT |
| Hu-Col4 | Forward | AGGTGTTGACGGCTTACCTG |
|  | Reverse | TTGAGTCCCGGTAGACCAAC |
| Hu-Col1 | Forward | GTGATGCTGGTCCTGTTGGT |
|  | Reverse | CACCATCGTGAGCCTTCTCT |
| ED-A | Forward | CCAGGTACAGGGTGACCTAC |
|  | Reverse | CTCTCCATATCATCGTGCAA |
| ED-B | Forward | CGCTAAACTCTTCCACCATT |
|  | Reverse | CCGCCATTAATGAGAGTGAT |
| IIICS | Forward | CCAGAGATCTTGGATGTTCC |
|  | Reverse | GCCTAAAACCATGTTCCTCA |
| CD24 | Forward | TCTAAATGTGGCTATTCTGTC |
|  | Reverse | TATTTGGGAAGTGAAGACTGGA |
| CD44 | Forward | TCCAACACCTCCCAGTATGA |
|  | Reverse | GGCAGGTCTGTGATGT |
| ALDH1A1 | Forward | TGTTAGCTGATGCCGACTTG |
|  | Reverse | TTCTTAGCCCGCTCAACACT |
| ALDH1A2 | Forward | CTGGCAATAGTTCGGCTCTCTC |
|  | Reverse | TGATCCTGCAAACATGCTC |
| ALDH1A3 | Forward | TCTCGACAAAGCCCTGAAGT |
|  | Reverse | TATTCGGCCAAAGCGTATTC |
| OCT4 | Forward | ACCGAGTGAGAGGCAACC |
|  | Reverse | TGAGAAAGGAGACCCAGCAG |
| KLF4 | Forward | TACCAAGAGCTCATGCCACC |
|  | Reverse | TCATCTGAGCGGGCGAATTT |
| SOX2 | Forward | CGAGTGGAAACTTTTGTCGGA |
|  | Reverse | TGTGCAGCGCTCGCAG |
| NANOG | Forward | CTGCCGTCTCTGGCTATAGATAA |
|  | Reverse | TACGAATACATCTTCATCACCAA |
| GAPDH | Forward | GAGTCCAGGGCGTCTTCA |
|  | Reverse | GGGGTGCTAAGCAGTTGGT |

**Table S3. RNA-Seq analysis of siCtrl- or siNSDHL MCF-7-spheroids**

|  | **siNSDHL/siCtrl** | |
| --- | --- | --- |
|  | **Fold Change** | ***P* value** |
| NANOG | 0.357 | 0.131 |
| SOX2 | 0.101 | 0.066 |
